# Long Non-coding RNA *MEG3* Promotes Renal Tubular Epithelial Cell Pyroptosis by Regulating the miR-18a-3p/GSDMD Pathway in Lipopolysaccharide-Induced Acute Kidney Injury

**DOI:** 10.3389/fphys.2021.663216

**Published:** 2021-04-29

**Authors:** Junhui Deng, Wei Tan, Qinglin Luo, Lirong Lin, Luquan Zheng, Jurong Yang

**Affiliations:** The Third Affiliated Hospital of Chongqing Medical University, Chongqing, China

**Keywords:** acute kidney injury, renal tubular epithelial cells, pyroptosis, *MEG3*, GSDMD, sepsis, miR-18a-3p

## Abstract

**Background and Objective:** Acute kidney injury (AKI) is a complication of sepsis. Pyroptosis of gasdermin D (GSDMD)-mediated tubular epithelial cells (TECs) play important roles in pathogenesis of sepsis-associated AKI. Long non-coding RNA (lncRNA) maternally expressed gene 3 (*MEG3*), an imprinted gene involved in tumorigenesis, is implicated in pyroptosis occurring in multiple organs. Herein, we investigated the role and mechanisms of *MEG3* in regulation of TEC pyroptosis in lipopolysaccharide (LPS)-induced AKI.

**Materials and Methods:** Male C57BL/6 mice and primary human TECs were treated with LPS for 24 h to establish the animal and cell models, respectively, of sepsis-induced AKI. Renal function was assessed by evaluation of serum creatinine and urea levels. Renal tubule injury score was assessed by Periodic acid-Schiff staining. Renal pyroptosis was assessed by evaluating expression of caspase-1, GSDMD, and inflammatory factors IL-1β and IL-18. Cellular pyroptosis was assessed by analyzing the release rate of LDH, expression of IL-1β, IL-18, caspase-1, and GSDMD, and using EtBr and EthD2 staining. *MEG3* expression in renal tissues and cells was detected using RT-qPCR. The molecular mechanisms of *MEG3* in LPS-induced AKI were assessed through bioinformatics analysis, RNA-binding protein immunoprecipitation, dual luciferase reporter gene assays, and a rescue experiment.

**Results:** Pyroptosis was detected in both LPS-induced animal and cell models, and the expression of *MEG3* in these models was significantly up-regulated. *MEG3*-knockdown TECs treated with LPS showed a decreased number of pyroptotic cells, down-regulated secretion of LDH, IL-1β, and IL-18, and decreased expression of GSDMD, compared with those of controls; however, there was no difference in the expression of caspase-1 between *MEG3* knockdown cells and controls. Bioinformatics analysis screened out miR-18a-3P, and further experiments demonstrated that *MEG3* controls GSDMD expression by acting as a ceRNA for miR-18a-3P to promote TECs pyroptosis.

**Conclusion:** Our study demonstrates that lncRNA *MEG3* promoted renal tubular epithelial pyroptosis by regulating the miR-18a-3p/GSDMD pathway in LPS-induced AKI.

## Introduction

Sepsis, a syndrome characterized by systemic inflammation caused by infection, has become a global health problem (Singer et al., 2016; Rhodes et al., 2017). Acute kidney injury (AKI) is one of the most common complications in patients with sepsis (Bouchard et al., 2015). Septic patients with AKI show a 6-day longer hospital stay and 3.4-fold increased risk of mortality compared with those of septic patients without AKI (Søvik et al., 2019). The mechanism of sepsis-related AKI is complicated and poorly understood at the clinical and research levels (Gyawali et al., 2019). Therefore, more studies are needed to help diagnose and prevent sepsis-related AKI.

Renal tubular epithelial cells (TECs) are the main cell type in kidney tissue; therefore, programmed death of these cells is the main pathophysiological process of AKI (Krautwald and Linkermann, 2014). Studies have shown that pyroptosis of TECs plays an important role in the pathogenesis of sepsis-related AKI (Ye et al., 2019; Zhou et al., 2020). The classic pathway of pyroptosis involves inflammasome-mediated activation of caspase-1, which leads to the cleavage of gasdermin D (GSDMD) into the active *N*-terminal and formation of a cell membrane pore; these events promote the production and release of pro-inflammatory mediators, such as IL-1β and IL-18, thereby producing a waterfall-level inflammatory response (Shi et al., 2015; Broz and Dixit, 2016). The active *N*-terminal of GSDMD is an indispensable “molecular switch” required to open membrane pores and release inflammatory factors. Indeed, stimulation with lipopolysaccharide (LPS) cannot effectively promote the release of IL-1β, IL-18, and other pro-inflammatory mediators after a GSDMD knockout (Sborgi et al., 2016). Moreover, a genetic knockout of GSDMD can significantly improve renal function and alleviate renal tissue inflammation in AKI mice (Miao et al., 2019). Therefore, it is particularly important to explore the regulatory mechanisms of GSDMD in TEC pyroptosis during sepsis-related AKI.

Long non-coding RNAs (lncRNAs), which are RNAs having a length of more than 200 nucleotides, are involved in the regulation of various biological processes such as the cell cycle, apoptosis, and pyroptosis (Mahmoud et al., 2019; He et al., 2020; Simion et al., 2020). LncRNA maternally expressed gene 3 (*MEG3*), an imprinted gene related to tumorigenesis (Braconi et al., 2011; He et al., 2017), is involved in pyroptosis occurring in multiple organs, such as that occurring during hyperbaric oxygen lung injury (Zou et al., 2020), atherosclerotic endothelial injury (Zhang Y. et al., 2018), or cerebral ischemia/reperfusion injury (Liang et al., 2020). *MEG3* shows up-regulated expression in renal tissue and renal TECs of patients with AKI (Yang et al., 2018). However, the role of *MEG3* in TEC pyroptosis occurring during sepsis-related AKI is unknown. In this study, we used *in vivo* and *in vitro* models to examine the role and mechanisms of *MEG3* in pyroptosis occurring during LPS-induced AKI. The findings obtained in our present study will provide new targets for the clinical diagnosis and prevention of septic AKI.

## Materials and Methods

### Mouse Model

Adult male C57 mice, weighing about 22–28 *g*, were purchased from the Animal Experimental Center at the Chongqing Medical University. Mice were randomly divided into four groups and injected intraperitoneally with normal saline or LPS (Sigma-Aldrich, United States) that was diluted with saline to the concentrations of 10, 20, and 40 mg/kg. Blood and renal tissues were collected at 24 h after the injection. For specimen collection, the mice were anesthetized by intraperitoneal injection with sodium pentobarbital; then, 1.5 ml blood was collected from the eye orbit, and the thoracic cavity was opened for cardiac perfusion. Renal-tissue specimens were obtained from both kidneys, the kidney capsule was removed, and part of the kidney was fixed in 4% paraformaldehyde for renal pathological tissue staining; the remaining part was stored at –80°C for subsequent analyses.

### Cell Model

Human primary renal TECs and epithelial cell medium were purchased from Sciencell, United States. Epithelial cells were cultured in a constant-temperature incubator maintained at 37°C and 5% CO_2_, in an epithelial cell medium containing 2% fetal bovine serum, 1% epithelial cell growth factor, and 1% penicillin/streptomycin; cells within four generations were used for experiments. When the cells were at 90% confluence, PBS (used as vehicle control) or LPS (diluted in PBS to the concentrations of 15, 30, or 60 μg/ml) was added into the culture medium. Cells and cellular supernatants were collected after continuous culture for 24 h.

### Assessment of Renal Function

Collected blood was stored in a refrigerator at 4°C for 1 h, then centrifuged at 4°C in a low-temperature centrifuge (at 3,000 rpm for 10 min); supernatants were then collected and used for the detection of serum creatinine and urea nitrogen levels using a Beckman 5800 automatic analyzer (Beckman, Germany).

### PAS Staining

Renal tissues were fixed overnight in 4% paraformaldehyde, then washed in distilled water, dehydrated, embedded in paraffin, and sectioned at 4 μm, and then soaked in anhydrous ethanol, 95% ethanol, and 70% ethanol, for 5 min in each. To observe the degree of renal pathological injury, Periodic acid-Schiff (PAS) staining was performed as follows. The sections were immersed in periodate oxidation solution for 10 to 20 min, then washed twice with distilled water, stained with Schiff solution for 10 to 30 min at 37°C, and washed again with distilled water for 5 min. Nuclei were stained with hematoxylin for 3 to 5 min, differentiated in hydrochloric-acid alcohol, and washed with tap water until the nuclei turned blue. The sections were then dehydrated, and sealed with Permount^TM^ Mounting Medium. Two nephrologists, under double-blinded conditions, observed, and scored each specimen using a light microscope at 200×, and each specimen was randomly observed using at least 10 non-repetitive fields. The extent of tubular injury was assessed by estimating the percentage of renal tubular epithelial tissue necrosis as follows: 0 points for no injury; 1 point (0–10%) and 2 points (11–20%) for mild injury; 3 points (21–40%) and 4 points (41–60%) for moderate injury; and 5 points (61–75%) and 6 points (>75%) for severe injury.

### Western Blotting

After protein was extracted from renal tissues and cells, protein concentration was determined using the BCA method. After denaturation, 30–80 μg protein was separated using 12% SDS-PAGE. Proteins were transferred to PVDF membranes using wet transfer. The membranes were blocked in 5% skim milk at room temperature for 1.5 h, and then incubated overnight at 4°C with the following primary antibodies specific for: IL-1β (1:1000, #12242, Cell Signaling, Shanghai, China); IL-18 (1:1000, ab71495, Abcam, Shanghai, China); caspase-1 (1:500, GTX14367, GeneTex, United States); GSDMD (1:500, orb390052, Biorbyt, Cambridge, United Kingdom); and β-actin (1:5000, AC004, ABclonal, Wuhai, China). The membranes were then washed and incubated at room temperature in the dark for 2 h with the following secondary antibodies: anti-mouse IgG (H + L, DyLight^TM^ 800 Conjugate, 1:15000, #5257, Cell Signaling, Shanghai, China) and anti-rabbit IgG (H + L, DyLight^TM^ 680 Conjugate, 1:15000, #5366, Cell Signaling, Shanghai, China). A two-color infrared fluorescence imaging system was used to visualize protein bands. Protein concentration in the bands was quantified using ImageJ and normalized against that of β-actin, used as loading control.

### RT-qPCR

Total RNA was extracted from kidney tissues and cells using Trizol, and RNA was quantified using OD 260 nm. cDNA reverse transcription and amplification were performed using the Hairpin-it^TM^ microRNA and U6 snRNA Normalization RT-PCR Quantitation Kit, or Custom gene qRT-PCR Quantitation Kit (GenePharma, Suzhou, China). Relative gene expression was assessed using the 2^–ΔΔ^ ^Ct^ formula, with U6 or GAPDH used as housekeeping genes. The primer sequences were as follows:

**Table:** 

Primer name	Base sequence
mmu-MEG3 (F)	5′ -CGCGAGGACTTCACGCACAA-3′
mmu-MEG3 (R)	5′ -TCTGTGTCCGTGTGTCCAGG-3′
hsa-MEG3 (F)	5′ -CTGCCCATCTACACCTCACG-3′
hsa-MEG3 (R)	5′ -ATCCTTTGCCATCCTGGTCC-3′
mmu-miR-18a-3P (F)	5′ -TCCAGACTGCCCTAAGTGC-3′
mmu-miR-18a-3P (R)	5′ -CAGTGCGTGTCGTGGAGT-3′
mmu-miR-541-3P (F)	5′ -ATCAGACTATGGCGAACACAGAA-3′
mmu-miR-541-3P (R)	5′ -TATCCTTGTTCACGACTCCTTCAC-3′
mmu-miR-654-5P (F)	5′ -TGGTAAGCTGCAGAACATGTGT-3′
mmu-miR-654-5P (R)	5′ -ATCCAGTGCAGGGTCCGAGG-3′
hsa-miR-18a-3P (F)	5′ -TGATACTGCCCTAAGTGCTCC-3′
hsa-miR-18a-3P (R)	5′ -CAGTGCCGTGTCGTGGAGT-3′
hsa-GSDMD (F)	5′ -TGATACTGCCCTAAGTGCTCC-3′
hsa-GSDMD (R)	5′ -GGCTCAGTCCTGATAGCAGTG-3′
mmu-GAPDH (F)	5′ -GAGAAACCTGCCAAGTATGATGAC-3′
mmu-GAPDH (R)	5′ -AGAGTGGGAAGTTGCTGTTGAAG-3′
hsa-GAPDH (F)	5′ -CATGAGAAGTATGACAACAGCCT-3′
hsa-GAPDH (R)	5′ -AGTCCTTCCACGATACCAAAGT-3′
mmu-U6 (F)	5′ -CAGCACATATACTAAAATTGGAACG-3′
mmu-U6 (R)	5′ -ACGAATTTGCGTGTCATCC-3′
hsa-U6 (F)	5′ -CAGCACATATACTAAAATTGGAACG-3′
hsa-U6 (R)	5′ -ACGAATTTGCGTGTCATCC-3′

### Lactate Dehydrogenase Release Rate (LDH%)

Cells were seeded in 96-well plates, and the wells were designated into the following groups: control wells, treatment wells, maximum-release wells, and spontaneous release wells. In the maximum-release group, 1 μl Triton X-100 was added to each well. The spontaneous release wells contained only cell-free media. After 30 min of continuous culture, supernatants were collected from all the wells and centrifuged at 4°C for 15 min. Then, 20 μl supernatant obtained from each well was added into the corresponding well of a new 96-well plate; 25 μl matrix solution and 5 μl coenzyme I application solution were then added to each well, and the plate was agitated in a water bath at 37°C for 15 min. Consequently, 25 μl 2, 4-dinitrophenylhydrazine was added to each well, and the plate was incubated in a water bath at 37°C for an additional 15 min. NaOH solution was diluted to 0.4 mol/l, and 250 μl of this diluted solution was added to each well and mixed by shaking. The plate was allowed to incubate at room temperature for 3 min, and LDH release rate was analyzed using a multifunctional enzyme label analyzer at 450 nm and calculated as follows: LDH release rate = (measured well OD value-spontaneous well OD value)/(maximum release well OD value-spontaneous well OD value) × 100%.

### ELISA

Cells were inoculated into 24-well plates, and cell-culture supernatants were collected after treatment. ELISA kits (Human IL-18 and IL-1β ELISA Kits from RayBiotech, United States) were used for detection. The standard-curve formula was determined using the OD value of the gradient concentration standard, and the final concentration of each well was calculated according to this formula.

### EtBr and EthD2 Staining

Two different red nucleic-acid dyes, EtBr (molecular weight of 394 Da) and EthD2 (molecular weight of 1293 Da), were used to detect membrane pores. TECs were seeded into the wells of a 24-well plate. After the TECs were treated, they were washed three times with PBS, fixed using 4% paraformaldehyde at room temperature for 15 min, and then washed again with PBS. The positive-control group was treated with PBST (PBS solution containing 1% Triton) and drilled at room temperature for 20 min. The cells were stained with DAPI nuclear stain in the dark for 5 min, and then treated with EtBr (25 μg/ml, Sigma, United States) or EthD2 (25 μg/ml, Thermo Fisher, United States). Images were acquired using a fluorescence microscope (Nikon, Japan) at 200×.

### Transfection of siRNA, Inhibitor and Mimic

siRNA, inhibitor and mimic were designed and synthesized by Shanghai Jima, China. Human renal TECs were transfected using Lipofectamine TM 3000 transfection reagent. Cells were used at 70% confluence. For transfection, siRNA, inhibitor, mimic, or transfection reagent was mixed with Opti-MEM^TM^ medium. The mixture containing siRNA, inhibitor or mimic was added to the mixture containing transfection reagent. After standing at room temperature for 15 min, each mixture was added to the cell medium. Subsequent treatments were administered at 48 h after transfection.

### Double Luciferase Reporter Assay

LncMEG3 wild-type and mutant plasmids, and GSDMD wild-type and mutant plasmids were constructed using a pMIRGLO vector. pMIRGLO-lncMEG3-WT and miR-18a-3p mimic, pMIRGLO-lncMEG3-WT and NC mimic, pMIRGLO-lncMEG3-Mut and miR-18a-3p mimic, pMIRGLO-lncMEG3-Mut and NC mimic, pMIRGLO-GSDMD-WT and miR-18a-3p mimic, pMIRGLO-GSDMD-WT and NC mimic, pMIRGLO-GSDMD-Mut and miR-18a-3p mimic, and pMIRGLO-GSDMD- Mut and NC mimic were co-transfected into human embryonic kidney cells (HEK293) using LipofectamineTM 3000. After 24 h, the cells were collected and lysed. Fluorescence activity was detected according to the instructions of the Dual-Luciferase^®^ Reporter Assay System (Promega, United States).

### RIP Assay

RNA immunoprecipitation (RIP) kit (BersinBiotech, Guangzhou, China) was used for RIP analysis. First, cultured primary human renal TECs were collected and lysed with a complete RIP lysis buffer. The cell lysate was then incubated overnight with magnetic beads (Abcam, Shanghai, China) containing either Ago2 antibody or negative control IgG antibody at 4°. The next day, the magnetic beads were washed with washing solution for 3 times, and then protease K buffer was used to remove the proteins. Finally, RNA was extracted for qRT-PCR.

### Statistical Analyses

IBM SPSS 23 statistical software was for data analysis; measurement data were expressed as mean ± standard deviation. One-way ANOVA was used after homogeneity of variance test for multiple-group comparison, and unpaired *t* test was used after normal distribution test for comparison between two groups. *P* < 0.05 indicated statistically significant difference.

## Results

### *MEG3* Is Up-regulated in the Kidneys of LPS-AKI Mice

**FIGURE 1:**
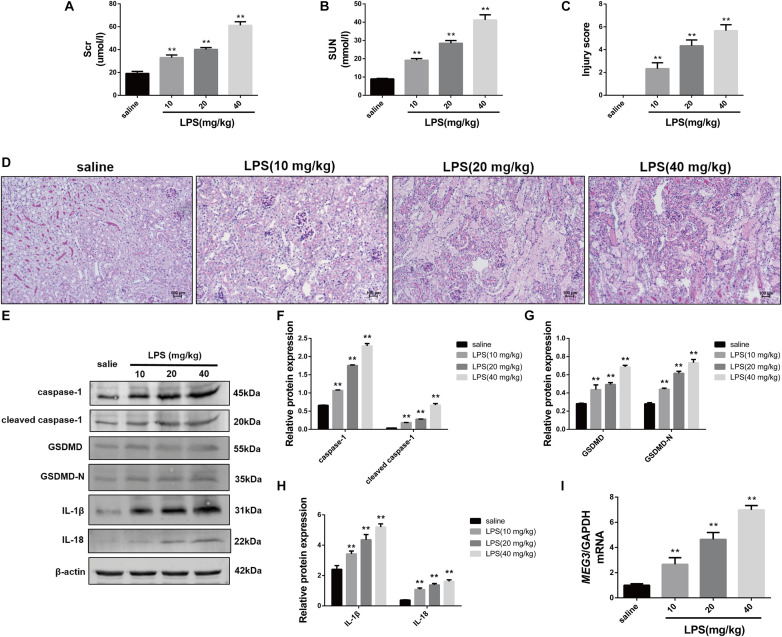
Increased expression of pyroptosis-associated proteins and *MEG3* in kidneys of mice with LPS-induced AKI (*n* = 6). **(A)** Serum creatinine level in mice with LPS-induced AKI and control saline-treated mice. **(B)** Serum urea nitrogen levels in mice with LPS-induced AKI and control saline-treated mice. **(C)** PAS staining of mouse kidneys (200×). **(D)** Score of renal tubular injury in mice. **(E–H)** Expression of caspase-1, cleaved caspase-1, GSDMD, GSDMD-N, IL-1β, and IL-18 in renal tissues of mice. **(I)** Expression level of *MEG3* mRNA in mouse kidney tissue. **P < 0.01, compared with normal saline-treated group; all values are expressed as mean ± standard deviation (SD).

First, we established our LPS-AKI mouse model to assess the expression levels of *MEG3* in the kidney. Compared with those in saline-treated mice, serum creatine and urea levels in LPS-treated mice had increased significantly (Figures 1A,B). Concurrently, PAS staining of renal tissue demonstrated that renal epithelial cells obtained from mice in the saline-treated group showed a close and orderly arrangement, with normal cell morphology, complete brush border, and clear lumen; in the LPS-treated group, we observed degeneration of the tubular epithelial vacuoloid, swelling, disappearance of the brush margin, tubular deposition, and infiltration of inflammatory cells. The renal-tubule injury score, obtained by scoring the degree of renal-tubule injury, was significantly increased in LPS mice compared with that of saline-treated mice (Figures 1C,D). These results show that renal function was impaired in mice after treatment with LPS. Next, we used western blotting to assess the expression levels of caspase-1, GSDMD, and inflammatory cytokines IL-1β and IL-18, in renal tissues during renal pyroptosis in mice. The expression levels of caspase-1, cleaved caspase-1, GSDMD, GSDMD-N, IL-1β, and IL-18 in the renal tissues of LPS-treated mice were significantly increased compared with those of the saline-treated mice (Figures 1E–H), indicating that renal pyroptosis occurred in mice after treatment with LPS. Using RT-qPCR, we also found that the level of *MEG3* in the LPS-treated group had increased in a concentration-dependent manner, consistent with the degree of renal damage and pyroptosis (Figure 1I). These results suggest that *MEG3* played a role in renal pyroptosis during septic AKI.

### *MEG3* Expression Is Up-Regulated in LPS-Induced Primary Human TECs

**FIGURE 2:**
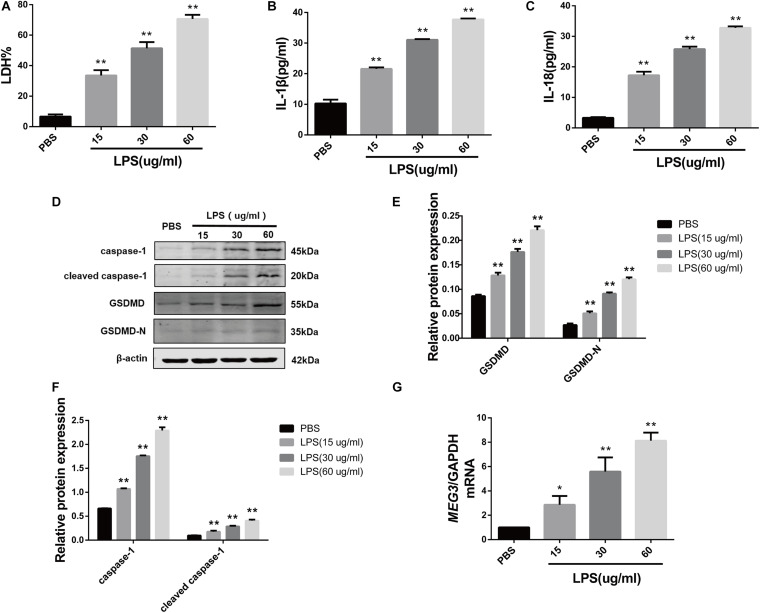
Increased expression of *MEG3* in primary human renal tubular epithelial cells treated with LPS. **(A)** LDH% in TECs supernatants. **(B,C)** Release of IL-1β and IL-18 in TECs supernatants. **(D–F)** Protein expression of caspase-1, cleaved caspase-1, GSDMD, and GSDMD-N in TECs. **(G)** Expression level of *MEG3* mRNA in TECs. *P < 0.05, **P < 0.01, and compared with the PBS group.

In order to delineate the relationship between MEG3 expression and TEC pyroptosis after LPS-induced injury, we established the model of LPS-induced injury in primary human TECs. Our results indicate that the levels of LDH in the supernatants of LPS-treated cells were significantly increased compared with those in PBS-treated controls (Figure 2A). The results of ELISA showed that the levels of inflammatory cytokines IL-1β and IL-18 in the supernatants of LPS-treated cells were also significantly increased compared with those in PBS-treated controls (Figures 2B,C). The results of our western blotting analysis showed that the levels of caspase-1, cleaved caspase-1, GSDMD, and GSDMD-N in LPS-treated cells were significantly increased compared with those of PBS-treated controls (Figures 2D–F). These findings indicate that pyroptosis occurred in primary human TECs after treatment with LPS. Similarly, RT-qPCR analysis showed that the levels of *MEG3* in the LPS-treated cells had increased in a concentration-dependent manner (Figure 2G). These results further suggest that *MEG3* may have a potential function in TEC pyroptosis after LPS-induced injury.

### Knockdown of *MEG3* Inhibits LPS-Induced Pyroptosis in Primary Human TECs

**FIGURE 3:**
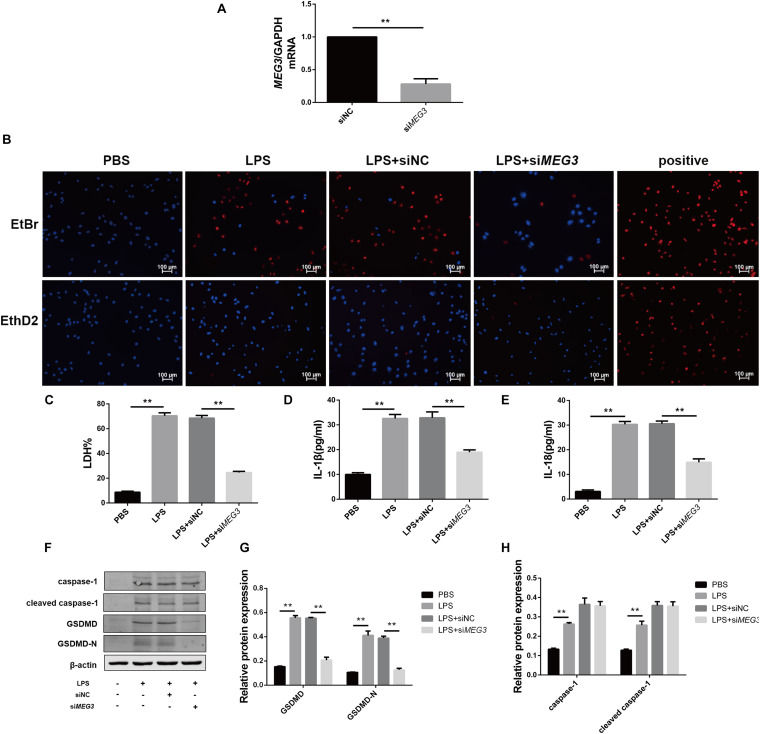
Knockdown of *MEG3* in TECs inhibited LPS-induced pyroptosis. **(A)** *MEG3* mRNA levels transfected with siRNA. **(B)** siRNA-transfected TECs stained with EtBr and EthD2. **(C)** LDH% in supernatants obtained from siRNA-transfected TECs. **(D–G)** Release of IL-1β and IL-18 in supernatants obtained from siRNA-transfected TECs. **(F–H)** Protein expression of caspase-1, cleaved caspase-1, GSDMD, and GSDMD-N in siRNA-transfected TECs. **P < 0.01, compared with the respective control group.

To verify the effect of *MEG3* on LPS-induced primary human TEC pyroptosis, we transfected *MEG3* siRNA into primary human TECs. Our results indicate that the relative mRNA level of *MEG3* in TECs transfected with si*MEG3* was reduced by approximately 73% compared with that in TECs transfected with NC siRNA (Figure 3A). Next, we detected pyroptosis using nucleic-acid dyes EtBr and EthD2 in in TECs transfected with si*MEG3* and in NC siRNA-transfected controls. During the process of pyroptosis, cells form a membrane pore having a diameter of approximately 20 nm. The nucleic-acid dye EtBr, which possesses small molecular weight, can pass through the membrane pore, but the large-molecular—weight EthD2 cannot. Therefore, cells positive for EtBr and negative for EthD2 staining are considered to be undergoing pyroptosis (Wang et al., 2017; Feng et al., 2018). Our results show that the number of pyroptotic TECs was significantly increased after treatment with LPS (60 μg/ml; the same concentration was used in subsequent experiments), but reduced after transfection with si*MEG3*, compared with that in the NC siRNA-transfected group (Figure 3B). The levels of LDH, IL-1β, and IL-18 were significantly reduced in the supernatants of siMEG3-transfected cells compared with those of NC siRNA-transfected group (Figures 3C–E). These results suggest that knockdown of *MEG3* in TECs could reduce LPS-induced pyroptosis. In si*MEG3*-transfected TECs, LPS induced a significant decrease in the expression of GSDMD precursor and active form, but no difference was observed in the expression of caspase-1 precursor and active form (Figures 3F–H). Based on these results, we speculate that the mechanism of *MEG3*-mediated promotion of pyroptosis may occur through regulation of GSDMD expression rather than regulation of its activation.

### miR-18a-3P Is Decreased in LPS-AKI

**FIGURE 4:**
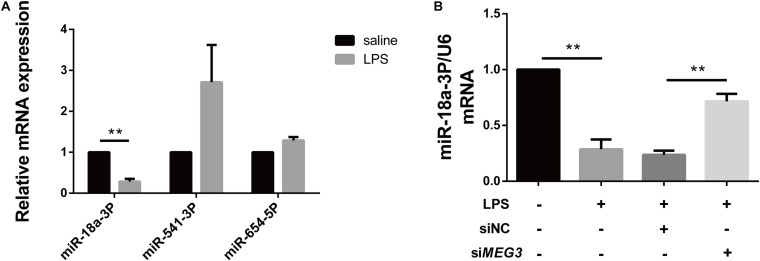
miR-18a-3P is decreased in LPS-AKI. **(A)** Expression levels of miRNA in renal tissues of LPS-AKI mice (40 mg/kg). **(B)** Expression levels of miR-18a-3p in siRNA-transfected TECs. **P < 0.01, compared with the respective control group.

Long non-coding RNAs are a newly discovered type of regulatory RNAs that competitively regulate the expression of target genes by interfering with miRNAs. To explore the mechanism of *MEG3*-mediated regulation of *GSDMD* expression, we used http://www.targetscan.org/vert_72/and http://carolina.imis.athena-innovation.gr/diana_tools/web/index.php?r=lncbasev2%2Findex to predict miRNAs regulated by *MEG3* and targeting *GSDMD* expression. Three miRNAs related to inflammation (miR-18a-3P, miR-541-3P, and miR-654-5P) were screened out using our functional review. We then assessed the expression of each miRNA in the renal tissues of our LPS-AKI mice. Our results indicate that only miR-18a-3P expression was significantly decreased (Figure 4A). We also found that miR-18a-3P expression was down-regulated in LPS-induced primary human TECs, but increased after transfection with si*MEG3* compared with that after transfection with siNC (Figure 4B). This result indicates that *MEG3* may regulate GSDMD through miR-18a-3P.

### Overexpression of miR-18a-3P Inhibits LPS-Induced Pyroptosis in Primary Human TECs

**FIGURE 5:**
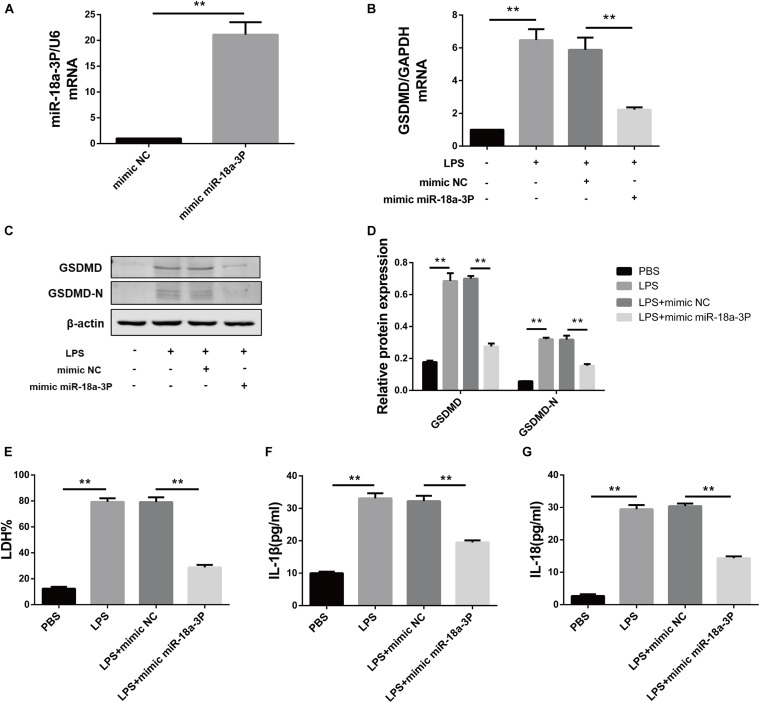
Overexpression of miR-18a-3P inhibits LPS-induced pyroptosis in primary human TECs. **(A)** Expression level of miR-18a-3p mRNA in TECs transfected with mimic. **(B)** GSDMD mRNA expression level in TECs transfected with mimic. **(C,D)** Protein expression levels of GSDMD and GSDMD-N in TECs transfected with mimic. **(E)** LDH% in supernatants obtained from TECs transfected with mimic. **(F,G)** Release of IL-1β and IL-18 in supernatants obtained from TECs transfected with mimic. **P < 0.01, compared with the respective control group.

In order to verify the effect of miR-18a-3P on LPS-induced primary human TEC pyroptosis, we transfected an miR-18a-3P mimic into primary human TECs. Compared with NC mimic, the relative mRNA expression level of miR-18a-3P increased by approximately 20-fold after transfection with a miR-18a-3P mimic (Figure 5A). We also found that the expression level of GSDMD in primary human TECs was significantly decreased in the miR-18a-3P mimic group after treatment with LPS compared with that of the NC mimic group (Figures 5B–D). At the same time, the levels of LDH, IL-1β, and IL-18 were significantly reduced in the cell supernatant after LPS treatment was also significantly reduced after transfection ofmiR-18a-3P mimic (Figures 5E–G). These results suggest that overexpression of miR-18a-3P inhibits LPS-induced pyroptosis in primary human TECs.

### Knocking Down miR-18a-3p Reversed the Inhibitory Effect of si*MEG3* on Pyroptosis in Primary Human TECs

**FIGURE 6:**
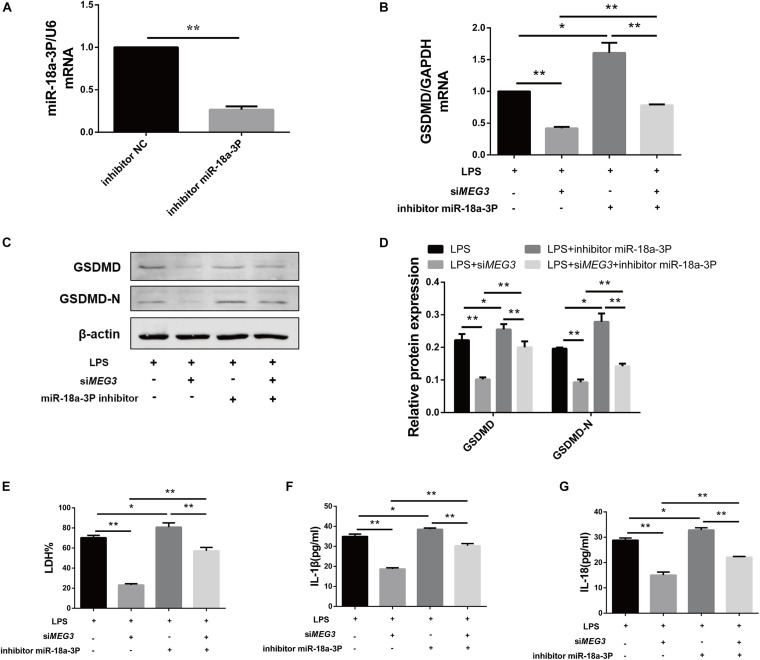
Knocking down miR-18a-3p reversed the inhibitory effect of siMEG3 on pyroptosis in primary human TECs. **(A)** Expression level of miR-18a-3p mRNA in TECs transfected with inhibitor. **(B)** GSDMD mRNA expression level in TECs transfected with siMEG3 and miR-18a-3P inhibitor separately or together. **(C,D)** Protein expression levels of GSDMD and GSDMD-N in TECs transfected with siMEG3 and miR-18a-3P inhibitor separately or together. **(E)** LDH% in supernatants obtained from TECs transfected with siMEG3 and miR-18a-3P inhibitor separately or together. **(F,G)** Release of IL-1β and IL-18 in supernatants obtained from TECs transfected with siMEG3 and miR-18a-3P inhibitor separately or together. *P < 0.05, **P < 0.01, and compared with the respective control group.

To verify whether knockdown of miR-18a-3p could reversed the inhibitory effect of si*MEG3* on pyroptosis in primary human TECs, we transfected si*MEG3* and miR-18a-3p inhibitor separately or simultaneously. First, we detected that the inhibitory efficiency of miR-18a-3P inhibitor reached 66% (Figure 6A). Then, we continued to detect that, miR-18a-3P inhibitor transfection significantly offset the decrease in GSDMD mRNA and protein levels caused by si*MEG3* transfection in LPS-induced primary human TECs (Figures 6B–D). Consistently, we also found that knocking down miR-18a-3P also significantly offset the reduction in LDH% and IL-1β and IL-18 release caused by si*MEG3* transfection (Figures 6E–G). These results indicate that *MEG3* regulates LPS-induced primary human TEC pyroptosis through miR-18a-3P.

### *MEG3* Binds to miR-18a-3P, and miR-18a-3P Binds to *GSDMD*

**FIGURE 7:**
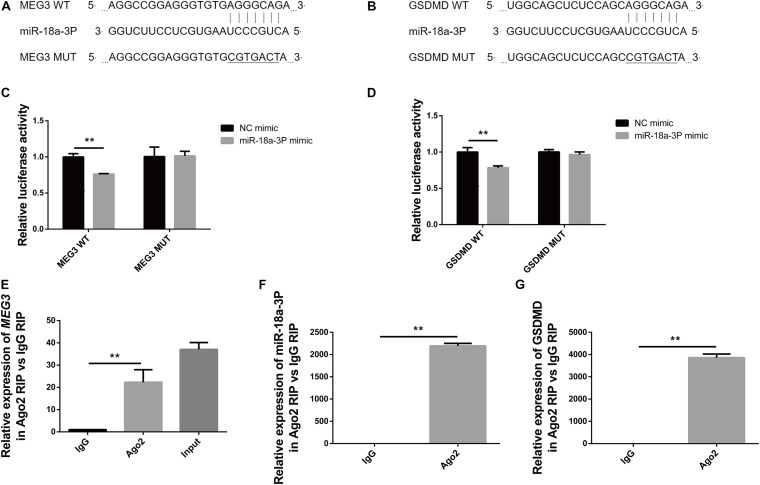
*MEG3* directly binds to miR-18a-3p, and miR-18a-3p directly binds to GSDMD. **(A)** Binding and mutation sites of *MEG3* and miR-18a-3p bases. **(B)** Binding and mutation sites of miR-18a-3p and GSDMD bases. **(C)** Dual-luciferase activity of *MEG3* and miR-18a-3p. **(D)** Dual luciferase activity of miR-18a-3p and GSDMD. **(E)** Relative expression of *MEG3* in RIP assay. **(F)** Relative expression of miR-18a-3p in RIP assay. **(G)** Relative expression of GSDMD in RIP assay. **P < 0.01, compared with the respective control group.

In order to detect the targeted binding relationships between *MEG3* and miR-18a-3P, and between miR-18a-3P and GSDMD, we constructed the pMIRGLO-lnc*MEG3*-WT, pMIRGLO-lnc*MEG3*-Mut, pMIRGLO-GSDMD-WT, and pMIRGLO-GSDMD-Mut plasmids, and used bioinformatics analysis to predict the base binding sites between *MEG3* and miR-18a-3P, and between miR-18a-3P and GSDMD (Figures 7A,B). Firstly, double-luciferase activity indicated that, miR-18a-3p mimic significantly inhibited the luciferase activity of *MEG3* and GSDMD wild-type carriers, but had no inhibitory effect on mutant carriers (Figures 7C,D). In addition, through RIP assay, it was found that *MEG3*, miR-18a-3P and GSDMD were all significantly up-regulated in Ago2-RIP compared with the negative control IgG-RIP (Figures 7E–G). These results indicate that *MEG3* can directly bind to miR-18a-3p and miR-18a-3p can directly bind to GSDMD.

## Discussion

In this study, we verified that pyroptosis occurred in both the *in vivo* and *in vitro* models of LPS-induced septic AKI. Our findings indicate that *MEG3* expression was up-regulated during pyroptosis. Our results, obtained using the *in vitro* model, show that *MEG3* played an important role in LPS-induced TEC pyroptosis, and that direct targeting of miR-18a-3p in regulation of GSDMD expression was involved in this pyroptotic mechanism.

Multiple mechanisms are involved in the development of septic AKI including renal hemodynamic abnormalities, inflammatory responses, and oxidative stress (Prowle and Bellomo, 2015; Shum et al., 2016; Khajevand-Khazaei et al., 2019). The inflammatory response is a major factor leading to impairment of renal function (Peerapornratana et al., 2019; Poston and Koyner, 2019). Studies on immune inflammatory cells have shown that pyroptosis, the most important cell-death pathway in septic AKI, produces pro-inflammatory mediators and triggers an inflammatory response (Doitsh et al., 2014). The activity of the inflammatory factor caspase-1 cleaves GSDMD, which exposes the GSDMD active *N*-terminal domain that inserts into the phospholipid structure of cellular intima, forming micropores of approximately 20 nm. This allows water to enter the cell, causing cell swelling and membrane rupture, and leading to the release of numerous pro-inflammatory mediators. This phenomenon is known as the classical pyroptotic pathway (Fink and Cookson, 2019). In previous studies, we examined indicators of pyroptosis in a rat model of ischemia/reperfusion AKI. Our results confirmed that pyroptosis occurs in renal TECs during AKI, which was closely related to AKI severity (Yang et al., 2014). Subsequent studies using mouse models of LPS-, cisplatin-, ischemia/reperfusion, and contrast-agent—induced AKI have reported that renal function and cell damage are considerably reduced by disruption of pyroptosis (Zhang Z. et al., 2018; Miao et al., 2019; Ye et al., 2019). In this study, we detected increased expression of caspase-1 precursor and its active form, GSDMD and its *N*-terminal, and inflammatory cytokines IL-1β and IL-18, in LPS-induced mouse kidneys and primary human TECs. Based on these findings, we conclude that pyroptosis is likely the main mechanism involved in pathogenesis of AKI.

Long non-coding RNAs, which are non-protein-coding RNAs, are involved in the regulatory mechanisms of various diseases (Zhang et al., 2017), including the injury and repair mechanisms of AKI (Liu Z. et al., 2019). The lncRNA *MEG3* was initially found to be a tumor suppressor that can inhibit the proliferation of tumor cells (Benetatos et al., 2011), but has also been found recently to regulate pyroptosis (Zhang Y. et al., 2018; Liang et al., 2020; Zou et al., 2020). Multiple studies have shown that *MEG3* is involved in regulating inflammatory-response mechanisms in various diseases such as acute pancreatitis (Xue et al., 2020), chronic obstructive pulmonary disease (Lei et al., 2021), and ultraviolet skin injury (Zhang et al., 2019). Disrupting the expression of *MEG3* can reduce the production of inflammatory factors via various pathways. In addition, *MEG3* also plays a key regulatory role in kidney diseases such as ischemic reperfusion-induced AKI (Yang et al., 2018), diabetic nephropathy (Zha et al., 2019), and transplanted kidney injury (Pang et al., 2019). In addition, one study has found that *MEG3* expression is increased in the blood of septic patients (Na et al., 2020). In this study, we detected significant upregulation of *MEG3* expression in both *in vivo* and *in vitro* models of septic AKI; this upregulation of *MEG3* expression occurred in a concentration-dependent manner and was consistent with pyroptosis. We detected LPS-induced TEC pyroptosis after transfection of si*MEG3*, and found that knockdown of *MEG3* reduced pyroptosis. Knockdown of *MEG3* also affected the expression of GSDMD precursor and *N*-terminal, but did not change the expression of caspase-1 precursor and active form. These findings indicate that regulation of *MEG3* in TEC pyroptosis occurred via regulation of GSDMD expression.

Long non-coding RNAs also function as ceRNAs and regulate other mRNA transcripts by competing for miRNAs, which can degrade target mRNAs and are, thereby, involved in regulation of gene expression (Song et al., 2016; Liu Y. et al., 2019). We used bioinformatics analysis to explore the specific miRNAs targeted by *MEG3* in regulation of GSDMD expression. Our results indicate that three miRNAs could be paired with both *MEG3* and GSDMD bases, and were functionally related to inflammation. We evaluated the expression of these miRNAs in the kidneys of our LPS mice, and found that only the expression of miR-18a-3p was negatively correlated with that of *MEG3*. Previous studies have reported that miR-18a-3p is down-regulated in the cruciate ligament of patients with osteoarthritis (Li et al., 2017). *In vitro* studies have shown that miR-18a-3p is involved in the regulation of pathological mechanisms of osteoarthritis (Ding et al., 2020). These studies suggest that miR-18a-3p plays a role in regulating inflammation. Therefore, we evaluated the role of miR-18a-3p in LPS-induced TEC pyroptosis. Our results show that knockdown of *MEG3* significantly increased miR-18a-3p expression in LPS-induced TECs, and that a miR-18a-3p mimic reduced the expression of GSDMD and the release of inflammatory factors. Moreover, our results also show that knocking down miR-18a-3P can reverse the inhibitory effect of si*MEG3* on pyroptosis in primary human TECs. Finally, we used a luciferase reporter gene and an RIP assay to evaluate the direct target-binding relationship between *MEG3* and miR-18a-3P, and between miR-18a-3P and GSDMD. Our results indicate that *MEG3* regulated GSDMD expression by acting as a ceRNA of miR-18a-3p.

In conclusion, we found that GSDMD-mediated TEC pyroptosis played an important role in the pathophysiological process of septic AKI. To the best of our knowledge, this study is the first to report that lnc can promote TEC pyroptosis by regulating the miR-18a-3p/GSDMD pathway. Our present study provides experimental and theoretical basis for further studies examining the mechanisms of renal TECs death in septic AKI, and provides a potential molecular target for clinical prevention and therapy. However, we only verified and explored the role and mechanism of *MEG3* in LPS-induced TEC pyroptosis. In our future studies, we will investigate this mechanism using additional *in vivo* models and clinical cases.

## Data Availability Statement

The original contributions presented in the study are included in the article/supplementary material, further inquiries can be directed to the corresponding author/s.

## Ethics Statement

The animal study was reviewed and approved by Biomedical Ethics Committee of Chongqing Medical University. Written informed consent was obtained from the owners for the participation of their animals in this study.

## Author Contributions

JD and JY designed the experiments. JY supervised the whole project. JD performed the major research and wrote the manuscript. WT, QL, LL, and LZ provided the technical support. All authors contributed to the article and approved the submitted version.

## Conflict of Interest

The authors declare that the research was conducted in the absence of any commercial or financial relationships that could be construed as a potential conflict of interest.
